# Cardiac Angiosarcoma as an Unexpected Cause of Palpitations: Case Report, Differential Diagnosis, and Current Therapeutic Approach to Cardiac Tumors

**DOI:** 10.7759/cureus.97085

**Published:** 2025-11-17

**Authors:** Wilmar A Ocampo Toro, Jaime H Alvarez Cuenca, María Azahara Hoyas García, Juan M Sanchez Bermejo, Calota Pardo Garcia

**Affiliations:** 1 Radiology, Hospital Universitario Severo Ochoa, Leganés, ESP

**Keywords:** cardiac angiosarcoma, cardiac magnetic resonance, cardiac radiology, echocardiography, palpitations, primary cardiac tumor

## Abstract

We present a case of an 83-year-old woman who presented with palpitations, asthenia, and exertional dyspnea. The electrocardiogram showed atrial flutter, and echocardiography (UCG) revealed a large mass occupying the right atrial chamber. Contrast-enhanced computed tomography (CECT) and cardiac magnetic resonance (CMR) confirmed its origin and extension into the superior vena cava (SVC). A multidisciplinary medical committee considered palliative surgery as the best therapeutic option for the patient due to the impossibility of a complete surgical resection of the tumor. Primary malignant neoplasms of the heart are extremely rare. Their clinical behavior is aggressive, with high morbidity and mortality and frequent surgical limitations due to location and extent at diagnosis. Imaging techniques are essential for diagnosis such as UCG for initial detection, CMR for tissue characterization, and CT or positron emission tomography-computed tomography (PET-CT) for staging and therapeutic planning. Definitive diagnosis requires histologic study, although it is not free of significant complications. This is a case report of a rare cardiac tumor followed by review of literature on its diagnosis and therapeutic approach.

## Introduction

Cardiac angiosarcoma is the most frequent primary malignant tumor of the heart, although its incidence is extremely low. In comparison, secondary cardiac neoplasms are 20-40 times more frequent, whereas pseudotumors (such as thrombi and anatomic variants) constitute the most common cause of a cardiac mass in clinical practice [1].

Clinical presentation is usually nonspecific, with symptoms such as dyspnea, tachycardia, or right heart failure, although some patients remain asymptomatic or mildly symptomatic. Transthoracic or transesophageal echocardiography (TTE and TEE, respectively) are the initial diagnostic methods due to their high sensitivity for detecting cardiac masses. Definitive diagnosis is histologic; however, cardiac biopsy carries a considerable risk of severe complications (hemorrhage and cardiac perforation) and a non-negligible false-negative rate due to tumoral necrosis and hemorrhage.

In this context, cardiac magnetic resonance (CMR) is the imaging modality of choice, as it allows differentiation of pseudomasses and benign tumors from malignant ones and provides detailed characterization using parameters such as size, morphology, mobility, involved chamber, and degree of infiltration. Contrast-enhanced computed tomography (CECT) and positron emission tomography-computed tomography (PET-CT) are primarily used to assess extracardiac extension and for staging, which are fundamental for therapeutic planning and prognosis.

Management of cardiac angiosarcomas is not standardized due to their low prevalence and aggressiveness. The most widely used treatment is radical surgery combined with adjuvant chemotherapy; however, outcomes are limited, with a median survival below 12 months in most patients.

The aim of this article is to elucidate the epidemiological, clinical, and imaging features of cardiac angiosarcoma, thereby facilitating accurate diagnostic and therapeutic strategies and preventing misdiagnosis with more common cardiac masses that generally carry a favorable prognosis. In contrast, cardiac angiosarcoma is distinguished by its extreme rarity, aggressive behavior, and poor clinical outcome.

## Case presentation

**Figure 1:**
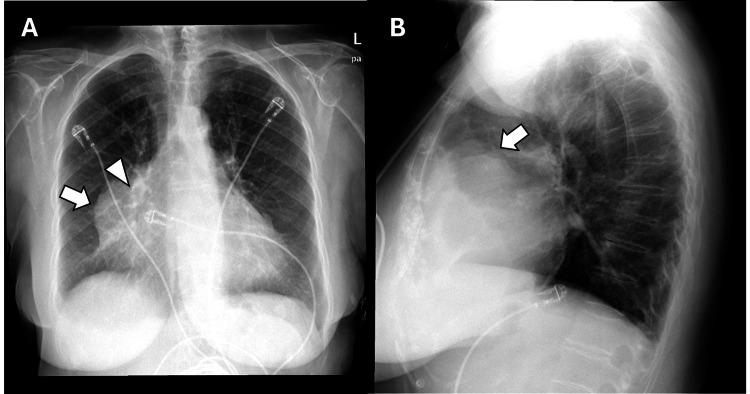
Posteroanterior (A) and lateral (B) chest radiographs. These radiographs show an increased cardiothoracic index secondary to a right paracardiac mass (arrow in A). There is blurring of the right heart contour, and on the contrary, the vessels and bronchi of the right pulmonary hilum can be seen (arrowhead in A), indicating that the mass affects the anterior mediastinum. On the lateral projection, the contour of the mass can be seen superimposed on the cardiac silhouette (arrow in B).

We report the case of an 83-year-old woman who presented with palpitations, asthenia, and dyspnea on moderate exertion. Her past medical history included arterial hypertension, hypercholesterolemia, and chronic venous insufficiency. A posteroanterior and lateral chest radiographs showed an increased cardiothoracic index due to a right paracardiac mass (Figure 1).

**Figure 2:**
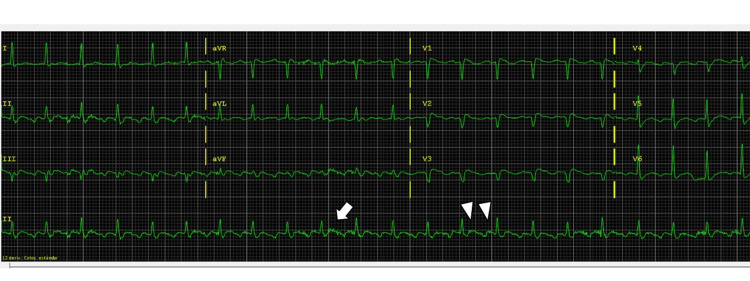
Electrocardiogram. Note the typical sawtooth appearance replacing the normal P waves (arrow on Lead II), which demonstrates atrial flutter in our patient. The electrocardiogram also shows that for every two P waves, only one QRS complex appears, revealing a 2:1 conduction (arrowheads). The T waves are difficult to see in this case due to their overlap with one of the P waves.

The electrocardiogram revealed atrial flutter at 220 beats per minute with 2:1 conduction (Figure 2).

**Figure 3:**
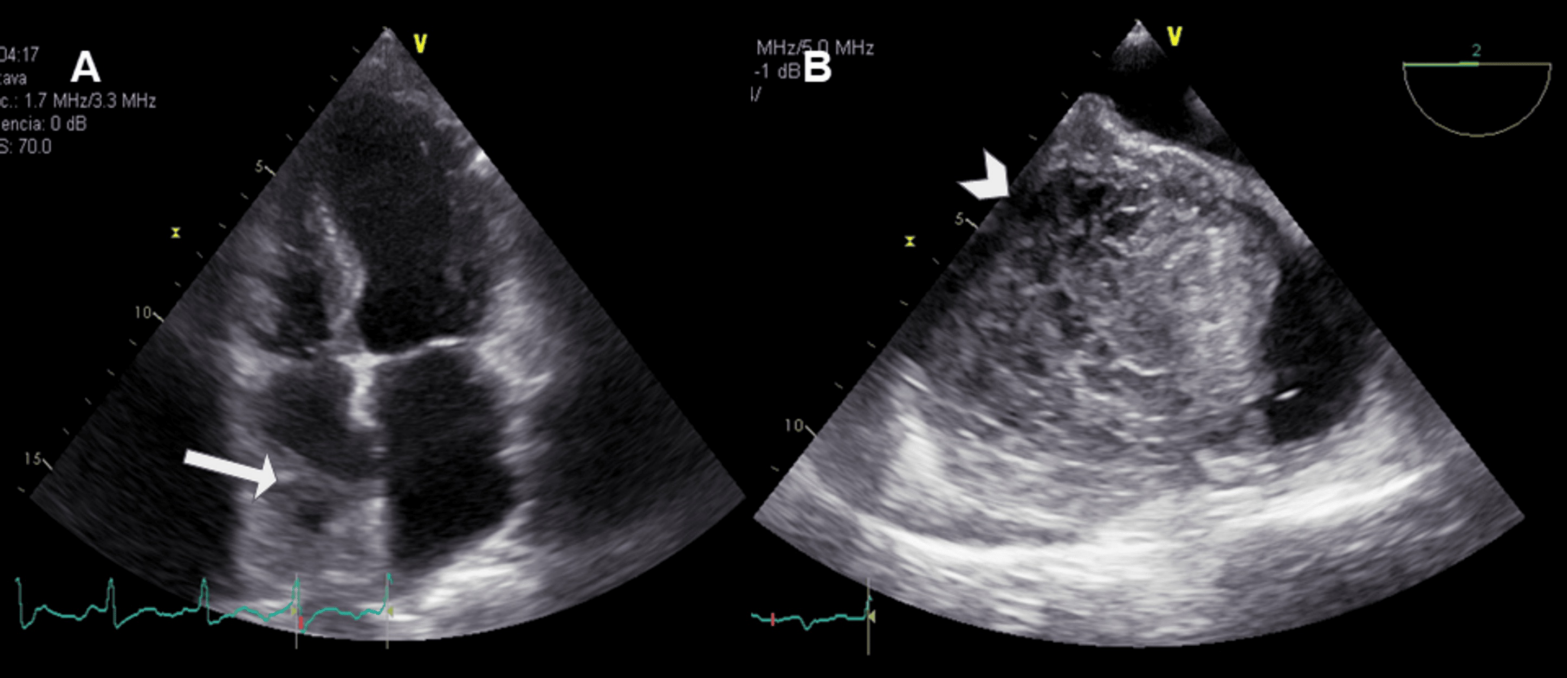
Echocardiographic studies. (A) TTE from an apical four-chamber view showed a mass of intermediate echogenicity occupying the right atrium. There is a hypoechoic center in the mass, suggesting an area of ​​necrosis (arrow). (B) TEE from a mid-esophageal view at the level of the left atrium, confirmed these findings, additionally showing that the mass originated from the lateral and posterior auricular walls with a broad base of implantation and heterogeneous echogenicity (arrow head). TTE: transthoracic echocardiography; TEE: transesophageal echocardiography.

On physical examination, there was a slightly elevated jugular venous pressure. Transthoracic echocardiography (TTE) showed a small amount of pericardial effusion and a mass occupying the right atrial chamber. TEE confirmed these findings, additionally showing that the mass originated from the lateral and posterior auricular walls with a broad base of implantation and heterogeneous echogenicity due to coexistence of anechoic and hyperechoic areas (Figure 3).

**Figure 4:**
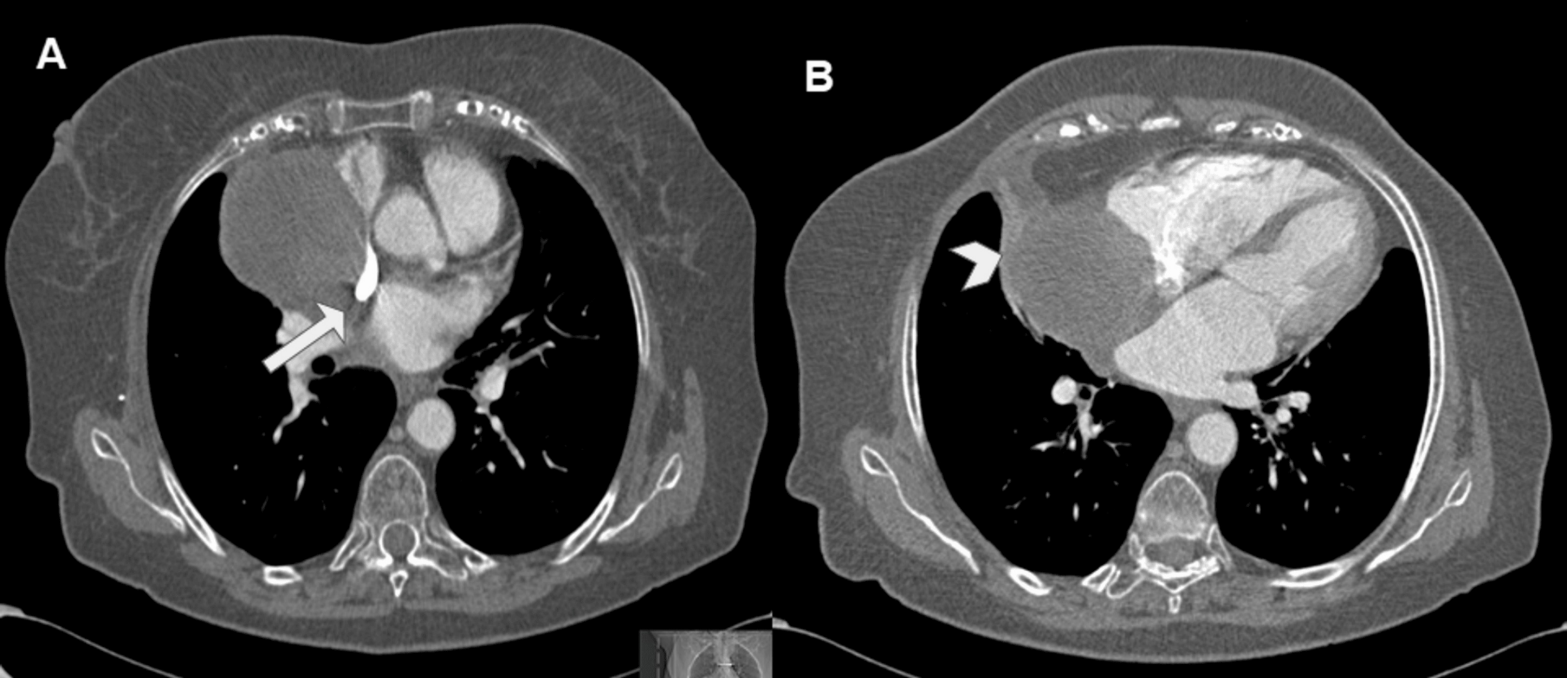
Contrast-enhanced CT in axial view at the four chambers (A) and at the SVC levels (B). (A) CT shows a voluminous rounded mass occupying the right atrial lumen. It seems to originate from the posterior and lateral auricular walls. (B) It also indented and reduced the caliber of SVC (arrow). The mass showed heterogeneous density, with soft-tissue areas with contrast uptake in its medial region and areas with fluid density in its lateral portion (arrowhead). CT: computed tomography; SVC: superior vena cava.

Given these findings, the patient was referred to our institution for further evaluation. CECT revealed a large, rounded mass occupying the right atrial lumen, with a maximum dimension of up to 7.5 cm (Figure 4).

**Figure 5:**
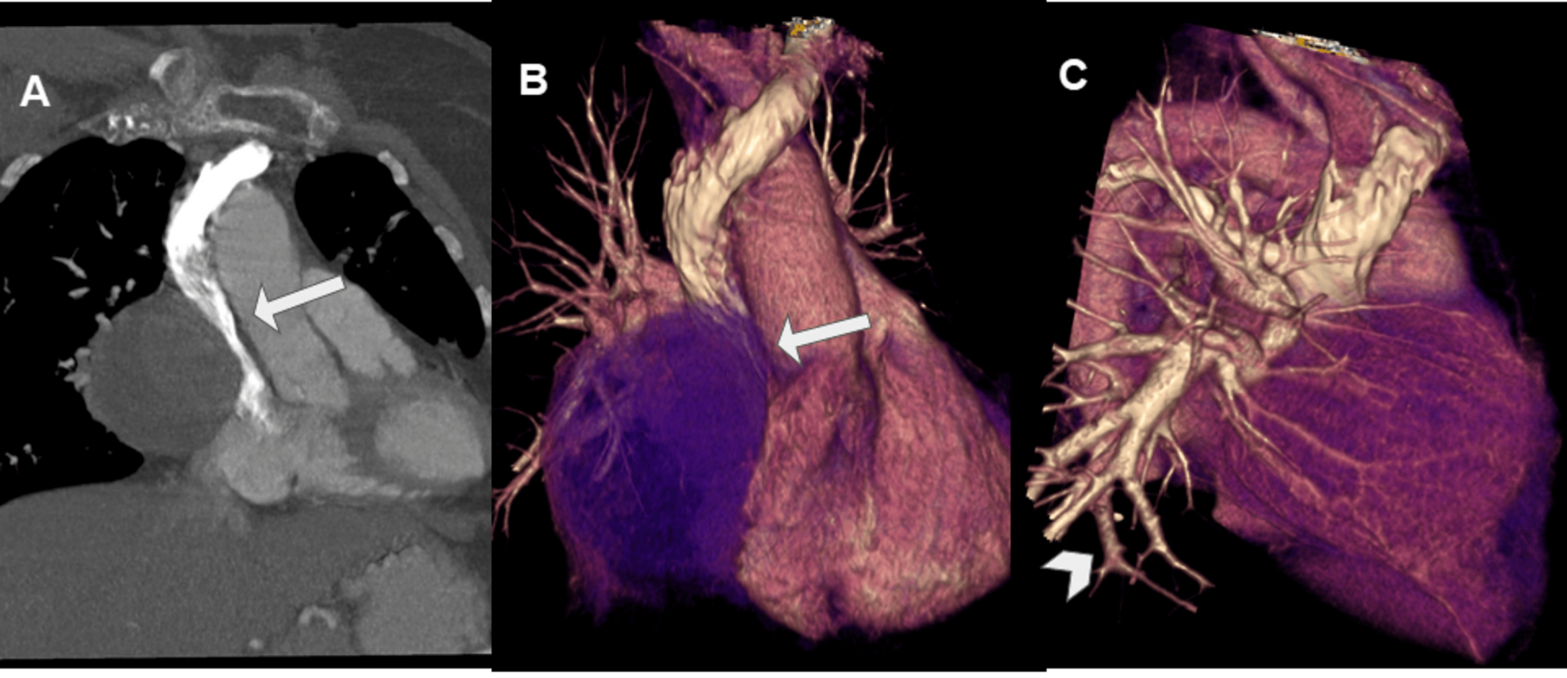
Multiplanar (A, coronal view) and 3D (B and C) reconstructions of the contrast-enhanced CT. These post-processed images were useful for eventual surgical planning, confirming the extrinsic compression of the SVC (arrow in A and B) and showing abundant feeding vessels of the tumor originated from the pulmonary artery (arrowhead in C). CT: computed tomography; SVC: superior vena cava.

It also indented and reduced the caliber of SVC (Figure 5).

The mass showed heterogeneous density: soft-tissue areas with contrast enhancement in the medial region and areas with fluid density in the lateral portion. No lymph node or distant organ involvement was revealed. CMR showed mild biventricular dysfunction (left ventricular ejection fraction (LVEF): 48% and right ventricular ejection fraction (RVEF): 46%) (Table 1).

**Table 1:** Functional parameters of left and right ventricles obtained by CMR imaging. Functional parameters of the left and right ventricles obtained by CMR, with reference values ​​for an 83-year-old woman with a body surface area of 1.6 m². CMR: cardiac magnetic resonance.

Parameter	Patient value	Reference range	Interpretation
LVEF			
Ejection fraction	48%	54–74%	Mildly reduced
End-diastolic volume	89.5 mL	84–145 mL	Normal
End-systolic volume	46.5 mL	28–58 mL	Normal
End-diastolic volume index	56.7 mL/m²	50–90 mL/m²	Normal
End-systolic volume index	29.4 mL/m²	15–40 mL/m²	Normal
Stroke volume	43.1 mL	55–90 mL	Slightly low
End-diastolic mass	39 g/m²	35–65 g/m²	Normal
Cardiac output	5.0 L/min	4.0–6.5 L/min	Normal
Cardiac index	3.17 L/min/m²	2.6–4.2 L/min/m²	Normal
RVEF			
Ejection fraction	46%	52–68%	Mildly reduced
End-diastolic volume	85.4 mL	80–150 mL	Normal
End-systolic volume	46.4 mL	32–70 mL	Normal
End-diastolic volume index	54.1 mL/m²	55–105 mL/m²	Low-normal
End-systolic volume index	29.4 mL/m²	20–45 mL/m²	Normal
Stroke volume	39.0 mL	55–85 mL	Mildly low
Stroke volume index	24.7 mL/m²	35–55 mL/m²	Reduced

**Figure 6:**
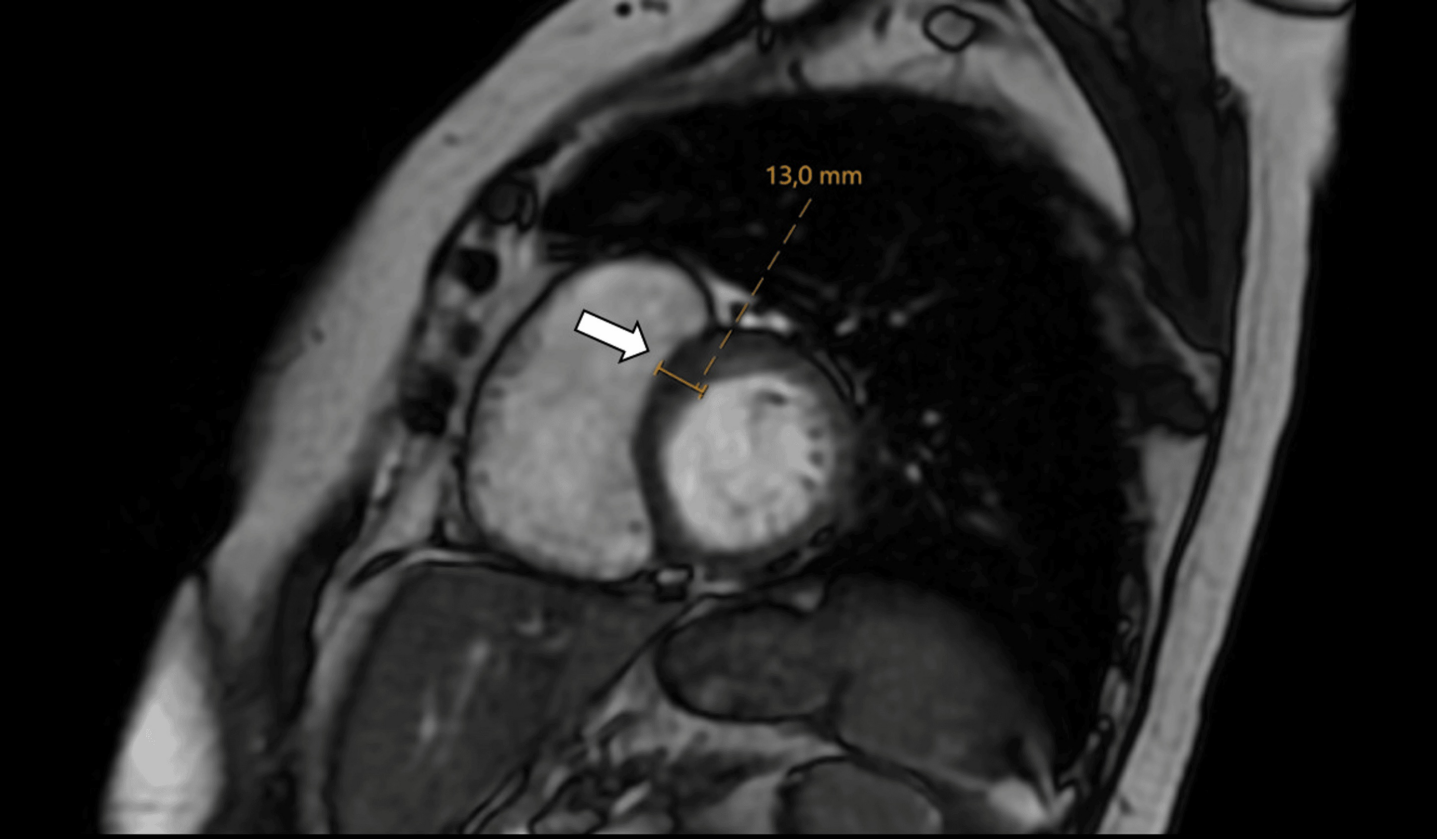
CMR imaging on steady-state free precession sequence in short axis. The short-axis image shows mild basal septal hypertrophy measuring 13 mm (arrow) in our patient. CMR: cardiac magnetic resonance.

It also demonstrated mild global ventricular hypokinesia, septal hypertrophy (13 mm), and moderate tricuspid regurgitation (Figure 6).

**Figure 7:**
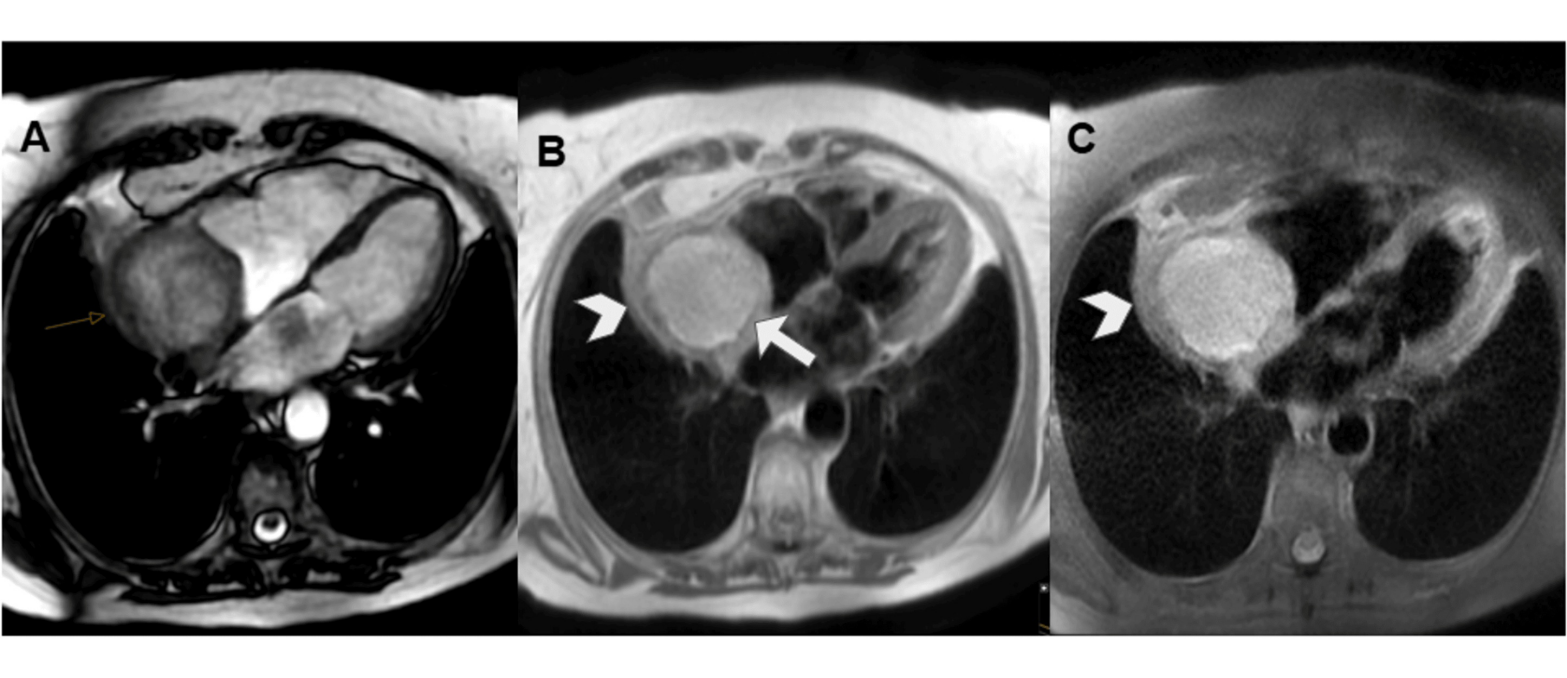
CMR imaging with steady-state free precession (A), T1-weighted turbo spin-echo (B), and T2-weighted turbo spin-echo with fat suppression sequences (C). These three sequences in an axial plane show a voluminous mass occupying the right atrium lumen. The mass showed heterogeneous signal with a central T1 hyperintense area, probably related to blood and/or proteinaceous content (arrow in B), and an irregular thickened peripheral wall that was slightly hypointense on T1 and slightly hyperintense on T2 (arrowhead in B and C). CMR: cardiac magnetic resonance.

It identified a mass up to 6 cm in diameter in the right atrium, with a broad base of implantation on its lateral and posterior walls, near the entry of the SVC, which it compressed. The inferior vena cava was unaffected. The mass showed heterogeneous signal intensity with a central hyperintense area on T1-wTSE sequences, probably related to blood and/or proteinaceous content, and a thickened irregular peripheral wall that was hypointense on T1-wTSE and hyperintense on T2-wTSE sequences (Figure 7).

**Figure 8:**
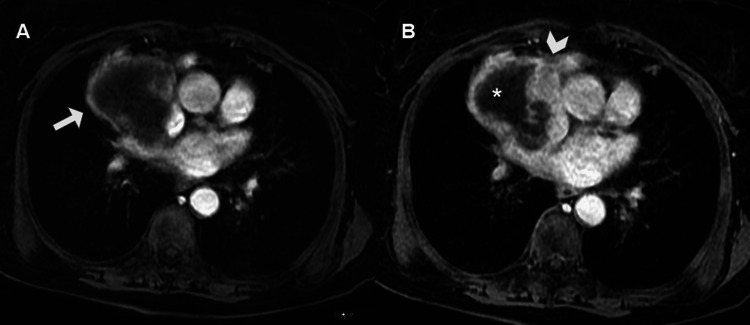
CMR imaging after gadolinium-based contrast administration with early enhancement at 2 minutes (A) and late enhancement at six minutes (B). The irregular wall showed intense contrast uptake on early phase (arrow in A) and on LGE depicted a nodular thickening at anteromedial aspect of the wall (arrowhead in B), the central tumoral area does not show any uptake contrast consisted with necrosis (asterisk in B). CMR: cardiac magnetic resonance; LGE: late gadolinium enhancement.

After gadolinium-based contrast administration, this wall showed intense contrast enhancement on first-pass perfusion at 20 seconds and nodular enhancement in its posteromedial aspect on late gadolinium enhancement (LGE) sequences (Figure 8).

Based on these radiologic characteristics and location, a cardiac sarcoma was suggested, with angiosarcoma being the leading diagnostic possibility by frequency. With this presumptive diagnosis, the patient was referred to a tertiary hospital for cardiac biopsy, which was non-diagnostic due to the presence of extensive tumor necrosis evidenced on CMR. A multidisciplinary team then decided on a diagnostic and palliative cytoreductive surgery, as the large size of the mass and involvement of the SVC made complete resection impossible. Histologic analysis confirmed the diagnosis of cardiac angiosarcoma.

Macroscopically, the sample had a hemorrhagic and necrotic appearance, with infiltration of the atrial wall and pericardium. Microscopically, a large proliferation of spindle-shaped endothelial cells with marked nuclear atypia, and frequent mitoses were observed. Irregular anastomosing vascular channels filled with blood and tumor cells were also observed. Immunohistochemistry revealed positivity for ERG, CD31, CD34, FLI-1, and factor VIII. ERG was a key marker for diagnostic confirmation.

Unfortunately, on the ninth postoperative day, the patient's condition deteriorated, presenting symptoms such as vomiting, pallor, and facial and upper-limb edema consistent with SVC syndrome, and was declared exitus within only several hours.

## Discussion

Primary cardiac tumors are very rare, with metastases being 20-40 times more prevalent than primary tumors [1,2]. Their incidence is 30 per 100,000 persons per year and their prevalence is 0.02-2.3% in autopsies [2-5]. Among primary neoplasms, 70-90% are benign. Classically, atrial myxoma has been considered the most frequent tumor, but recent studies suggest that papillary fibroelastoma is probably more frequent, underdiagnosed due to its small size and minimal clinical impact. In the pediatric population (patients under 16 years), the most frequent benign cardiac tumors are rhabdomyomas, teratomas, and fibromas.

Cardiac tumors are generally asymptomatic and are diagnosed incidentally by UCG or CT [1,2]. When symptoms occur, their severity is highly variable and depends on the size, location, and type of neoplasm. They can cause arrhythmias, obstructive symptoms, left ventricular dysfunction, heart failure and dyspnea, systemic or pulmonary embolism, and systemic symptoms such as weight loss and fever [1,4]. Cardiac biomarkers may be elevated but are nonspecific (Ca125, NSE) [6].

In clinical practice, the majority of cardiac masses are diagnosed by imaging studies such as UCG and CMR. However, the definitive diagnosis requires heart biopsy and histological analysis of the specimen [1]. Samples can be obtained endovascularly or percutaneously depending on location, or by open surgical biopsy [6]. Biopsy is nevertheless subject to a risk of potentially serious complications such as bleeding and cardiac perforation. In addition, for neoplasms with areas of necrosis or hemorrhage, it yields a high number of non-diagnostic results [6]. Furthermore, cytology of pericardial fluid is often negative for malignancy [6].

Therefore, imaging studies are essential for diagnosis. Both TTE and TEE are useful for initial detection [1,2,6,7]. Echo has high sensitivity for visualizing benign masses occupying the cardiac lumen and assessing their mobility and prolapse through valves (as occurs in myxomas). Masses can have heterogeneous or intermediate echogenicity (myxomas and fibromas) or can be hyperechogenic (typical of rhabdomyoma in children) [1,4]. 

CECT is useful for evaluating extracardiac and distant involvement in malignant tumors, as well as for assessing fat content (lipomas and lipomatous hypertrophy of the interatrial septum) and calcification (hemangiomas) of masses [1,4,7,8]. Cardiac CT with ECG gating can be useful when CMR is contraindicated, to identify intracavitary thrombi and to perform preoperative coronary assessment [7]. PET-CT is useful both for diagnosis and for distant staging; malignant tumors typically show higher fluorodeoxyglucose (FDG) uptake than benign ones [1].

CMR is the imaging modality of choice for cardiac neoplasms, as it is the most accurate for differentiating true neoplasms from pseudolesions such as anatomic variants or intracavitary thrombi, which are much more frequent. It also provides a high pre-test probability for distinguishing malignant from benign lesions and can even suggest different histologic subtypes [9]. This is due to its excellent tissue characterization and assessment of the relationship of the tumor with cardiac structures [1,4,7,8]. It also evaluates indirect tumor features such as location and association with pericardial and/or pleural effusion. Its role is heightened by the fact that cardiac biopsy carries potentially serious complications and a high false-negative rate in necrotic or hemorrhagic masses [2], as occurred in the presented case. CMR is also useful for preoperative planning and post-treatment follow-up.

The standard CMR protocol for the study of masses includes ECG-gated steady-state free precession (SSFP) cine sequences, also called “bright-blood,” which facilitate detection of intracavitary lesions and allow global assessment of cardiac function; morphologic and tissue-characterization sequences: T1- weighted turbo spin-echo (T1-wTSE), also called “black-blood” [1,7,9], and T2-weighted turbo spin-echo (T2-wTSE) sequences, which are used for distinguishing areas of necrosis and fat (which show signal suppression on fat-saturation sequences) and areas of hemorrhage; as well as post-contrast sequences with myocardial and fat nulling, both in early phases (first-pass perfusion) and in LGE from six minutes after contrast administration, which assess the degree of tumor vascularization and improve the detection of intracavitary thrombi (Table 2) [2].

**Table 2:** Standard CMR imaging protocol for the assessment of cardiac masses. CMR: cardiac magnetic resonance; LGE: late gadolinium enhancement.

Sequence type	Technique	Utility
Cine (“bright-blood”)	ECG-gated gradient-echo sequences	Detect intracavitary lesions Assessment global cardiac function
Morphologic (“black-blood”)	T1 turbo spin-echo T2 turbo spin-echo	Tissue characterization Identify necrosis, fat, and hemorrhage
Fat-saturation sequences	Applied to T1/T2	Confirm presence of fat within lesion
Post-contrast: early phase	First-pass perfusion with myocardial and fat nulling	Assessment of tumor vascularization
Post-contrast late phase (6 min)	LGE with myocardial and fat nulling	Improve detection of thrombi Evaluate degree/pattern of enhancement (helps differentiate thrombus vs tumor)

Symptomatic benign cardiac tumors or those with a high risk of complications (embolisms or arrhythmias) are treated surgically with complete resection, having an excellent prognosis and survival rate similar to that of the general population [1,2,7,8].

For primary malignant tumors at an early stage, treatment is surgical with complete resection, with the possibility of adjuvant chemotherapy or radiotherapy [2,3]. Treatment of advanced tumors consists of chemotherapy with the option of cytoreductive surgery, although the prognosis in these cases is poor, with a mean survival of nine to 16.5 months [7].

Cardiac pseudotumors: intracavitary thrombi and anatomical variants

Intracavitary thrombi

Thrombi are by far the most frequent cardiac mass, so distinguishing them from true tumors is a critical issue [7,8]. CMR is the most accurate technique to distinguish them, with the intravenous contrast sequence being essential for its visualization [2,8]. Their most frequent location is on the left atrium [1,8] (occurring in patients with atrial fibrillation and/or mitral valvulopathy, located on the roof or near the appendage), secondly in the left ventricle (in patients with ischemic heart disease, related to the akinetic segment, or in dilated cardiomyopathy). On the right atrium, they appear in patients with central venous catheterization or venous thromboembolic disease of the lower limbs.

**Figure 9:**
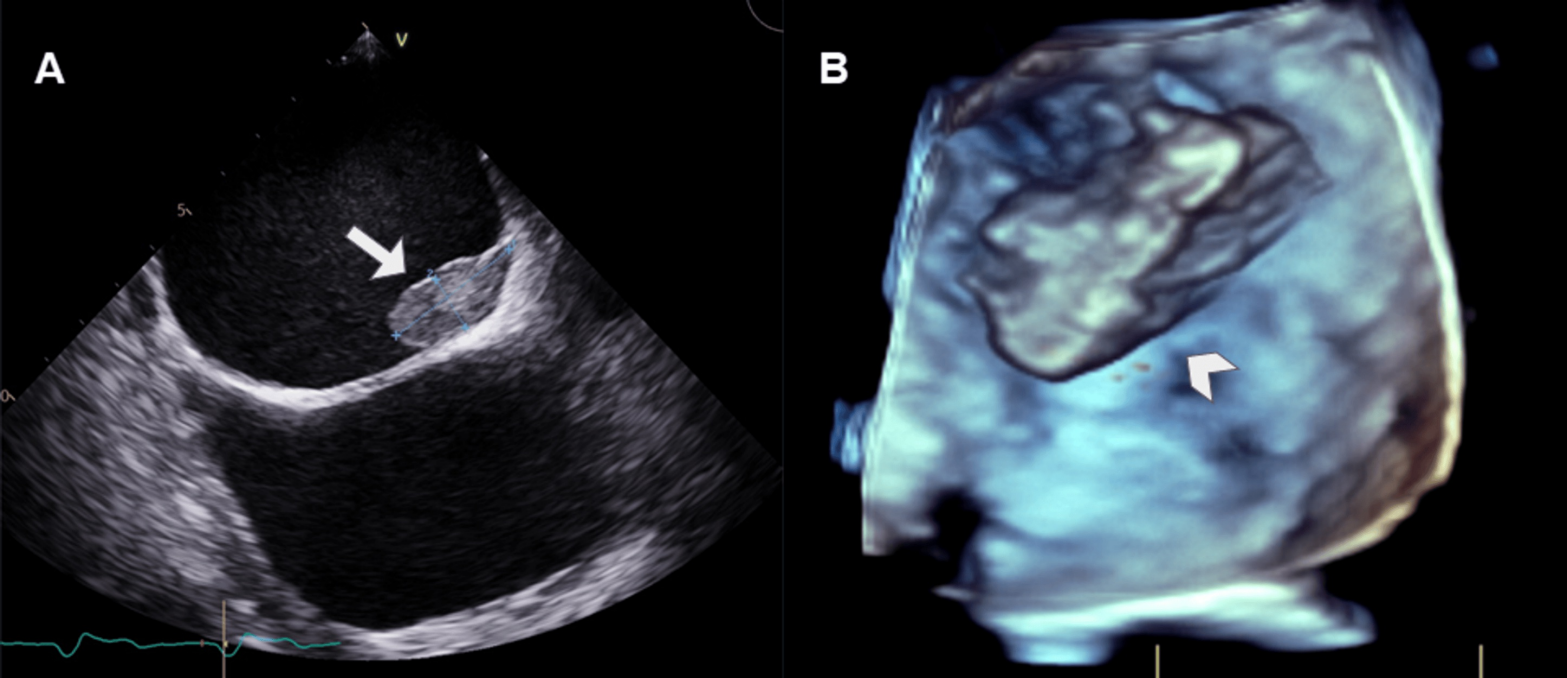
TEE in B mode (A) and 3D images in the left atrial (B). A woman presented with atrial fibrillation. TEE was performed and showed an ovoid lesion of intermediate echogenicity attached to the atrial roof (arrow in A). 3D echography images verify these findings (arrowhead in B) which are consistent with intracavitary thrombi. TEE: transesophageal echocardiography.

On imaging, they are lesions of round or elongated morphology with a broad base and less than 2 cm in size. They may be mobile or fixed in case of long evolution time [8]. On Echo, they may be hyperechoic or have intermediate echogenicity (Figure 9).

**Figure 10:**
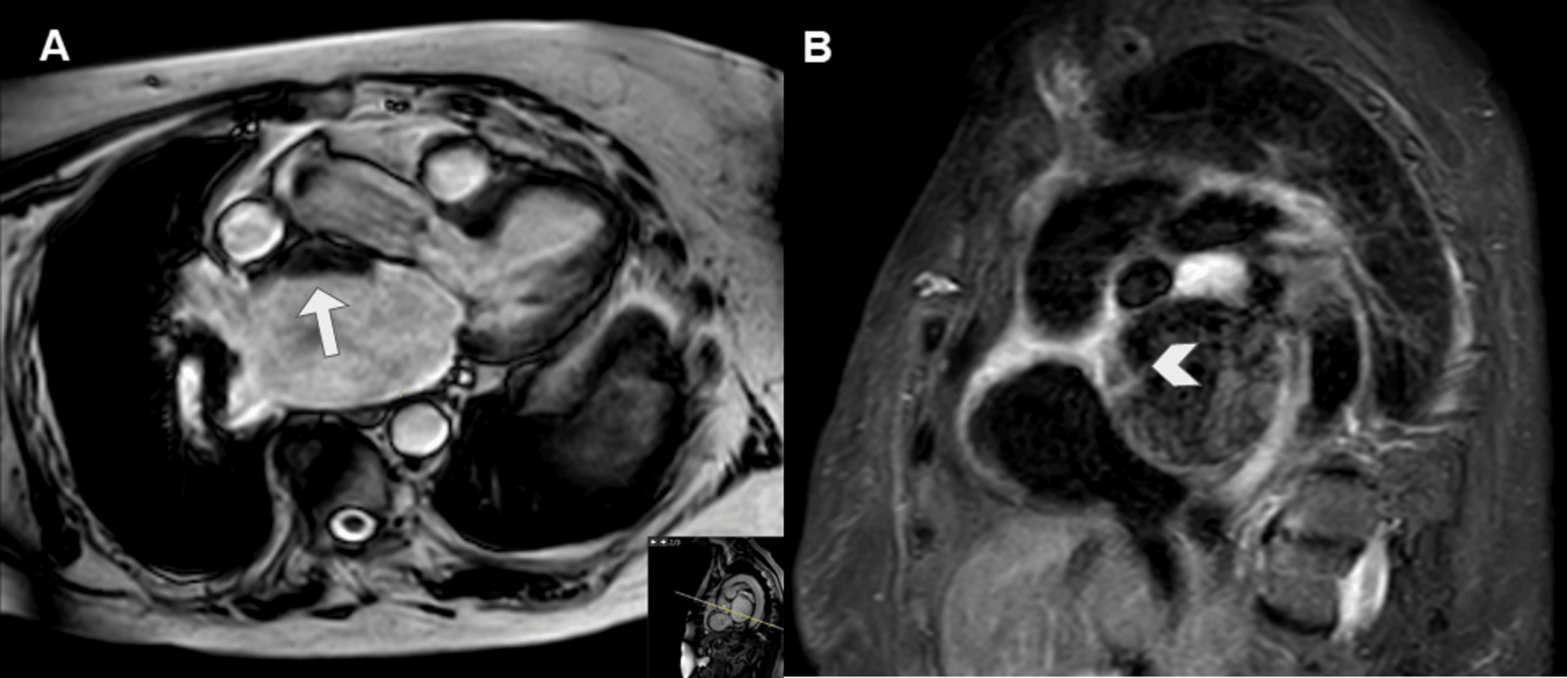
CMR imaging, cine sequences on three chambers (A) and STIR sequences on short axis (B). The images show an hypointense image in the roof of the left atrium (arrow in A).STIR sequences shows an hypointense lesion in the anterior portion of the atrial roof (arrowhead in B). CMR: cardiac magnetic resonance.

On CT, they are hyperdense with respect to the myocardium on non-contrast sequences and may show calcifications when chronic. On CMR, their signal varies according to their time of evolution, generally homogeneous: acute thrombi are hyperintense on T1- and T2-wTSE sequences, subacute are hyperintense on T1-wTSE and hypointense on T2-wTSE, and chronic are hypointense on both sequences (Figure 10) [4,8].

**Figure 11:**
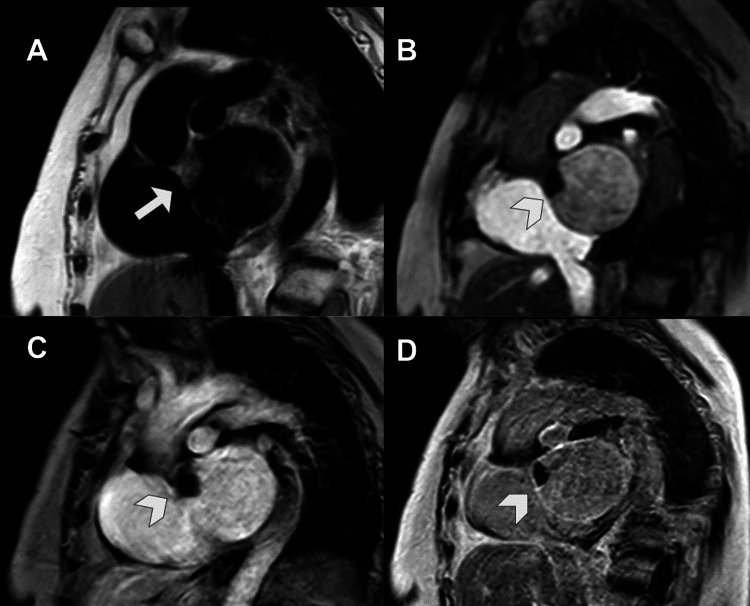
CMR imaging on T1 TSE (A) and T1 after administration of intravenous gadolinium perfusion (B), early enhancement (C) and LGE sequences (D). The images show an isointense image in the roof of the left atrium (arrow in A), which is hypointense in all sequences after contrast administration (arrowhead in B, C and D). Findings related to subacute atrial luminal thrombus. CMR: cardiac magnetic resonance; LGE: late gadolinium enhancement; TSE: turbo spin-echo.

After gadolinium administration, they show absence of enhancement in all modalities (perfusion, early enhancement at two minutes, and LGE) (Figure 11) [1,3,4,7,8].

Some chronic thrombi may show a thin peripheral uptake due to the development of a pseudocapsule. In cases of left ventricular thrombi, late-enhancement sequences without myocardial nulling (with fixed inversion times of 500 milliseconds) are useful, since the myocardium and the ventricular cavity appear hyperintense relative to the hypointense thrombus [9]. 

Crista Terminalis

It is a vertical band of myocardial tissue in the right atrium that runs from the SVC to the IVC, dividing its lateral from its posterior wall [4,7,9]. 

Eustachian Valve

Is is located at the level of the entry of the IVC. If prominent, it can also simulate thrombi in the right atrium [9].

Coumadin Ridge

It is a band of myocardial tissue in the left atrium that runs from the appendage to the left superior pulmonary vein [1,9]. 

Aberrant Papillary Muscle

It corresponds to a band of myocardium that runs from the free wall of the left ventricle to the basal segments of the interventricular septum [1].

Lipomatous Hypertrophy of the Interatrial Septum

**Figure 12:**
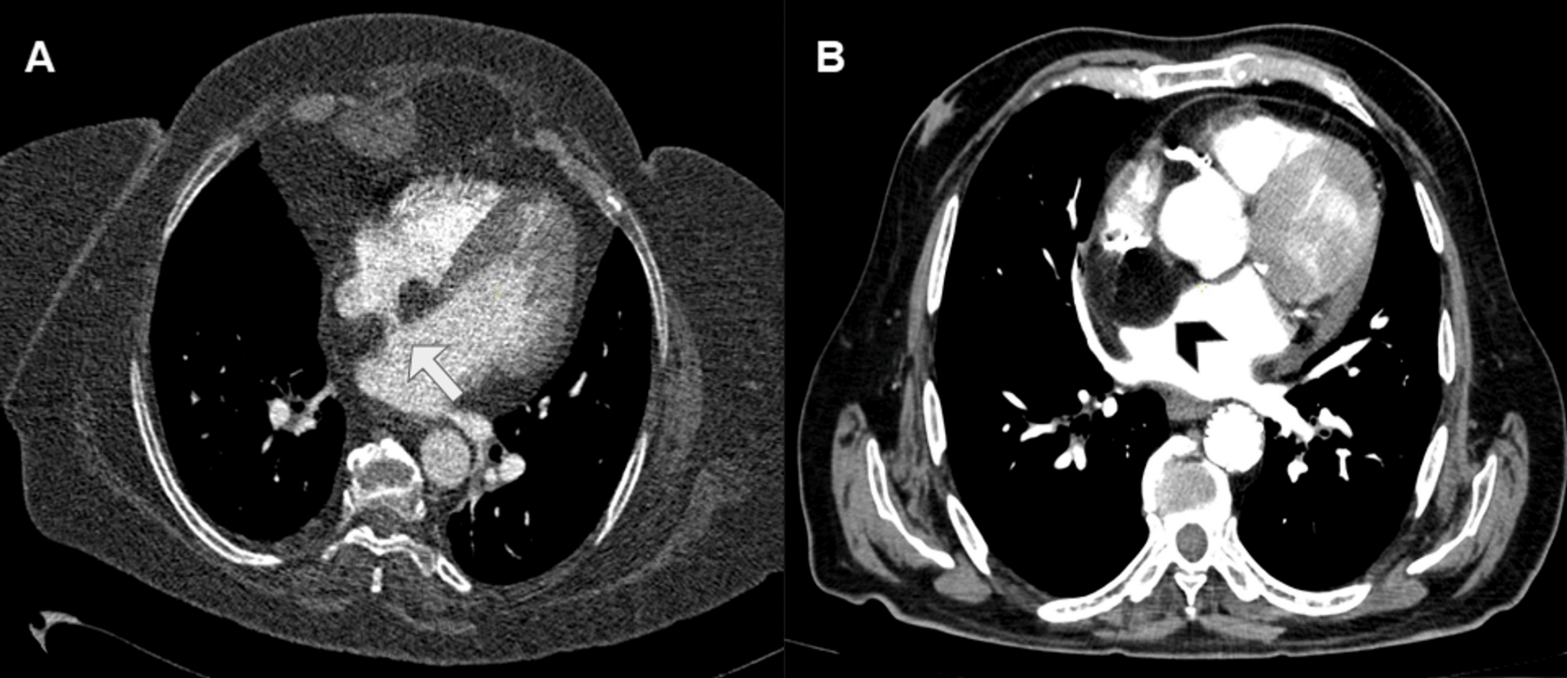
CECT in axial view in two different patients. CECT shows a lipomatous hypertrophy of the interatrial septum with a typical dumbbell or hourglass shape due to respect for the oval fossa and absence of capsule (arrow in A). Otherwise, lipomas in the interatrial septum do not spare the fossa ovalis and are encapsulated lesions, as is shown in B (arrowhead in B). CECT: contrast-enhanced computed tomography.

It is more frequent in women, obese patients, and those on chronic corticosteroid treatment [9]. It represents fatty hyperplasia of the interatrial septum of at least 2 cm in thickness, non-encapsulated and has continuity with epicardial fat. It has a characteristic morphology, by sparing the fossa ovalis, of an hourglass or dumbbell shape [1,7,9]. It shows signal characteristics identical to fat tissue of the rest of the body: hyperintense on T1- and T2-wTSE, suppressed with fat-saturation sequences, and no contrast enhancement. It may have areas of brown fat and therefore may show uptake on PET-CT. The difference from lipomas of the interatrial septum is that lipomas do not spare the fossa ovalis and are encapsulated (Figure 12) [1,2].

Other Pseudolesions

There are many other causes of cardiac masses that are beyond the scope of this paper. A few are worth mentioning, such as vegetations of both septic and aseptic origin, severe left ventricular hypertrophy with a mass-like appearance, pseudoaneurysms, or abscesses. In all of them, location, morphological characteristics, and clinical correlation are fundamental to distinguish them from true tumors.

Benign cardiac tumors

Papillary Fibroelastoma

It is the most frequent benign cardiac tumor found in autopsies, probably underdiagnosed due to its small size and absence of symptoms in almost all cases [1]. They are tumors that originate from the valvular endocardium in 80% of cases and from the endocardium of the cardiac chambers in 20% of cases [1,4].

They are small tumors (less than 1.5 cm and on average 2-7 mm) [4,7]. They are thought to derive from chronic injury or degenerative changes of endocardium tissue. There are cases of multiple lesions associated with radiotherapy, surgeries or rheumatic diseases, although they are much rarer. They have a rounded or elongated shape, generally pedunculated. Those with a valvular location arise from the surface of the valve leaflets opposite to the blood flow, most frequently from the aortic valve and the mitral valve [1,9].

In the few symptomatic cases, they cause embolisms and sudden death by obstruction of coronary flow [1,7]. Their diagnosis is made fundamentally with ETT and ETE due to their high sensitivity for their detection, followed by cardiac CT with cardiac synchronization due to its great spatial resolution. CMR can be useful to assess their fibrotic tissue, but they may go unnoticed owing to their small size and mobility. On CMR, they are sessile lesions with hypointense signal on T1-wTSE, isointense on T2-wTSE sequences, and hypointense on SSFP cine sequences, where they also show turbulence and may show homogeneous late contrast uptake on LGE sequences. On PET-CT, they may have moderate FDG uptake (SUV max of 3.1) [4]. Treatment is surgical if their size is more than 1 cm or if they cause symptoms, although some authors consider surgery whenever they are located in left-sided chambers due to the risk of systemic embolism [7]. Cases of recurrence have been described after surgical treatment, so echocardiographic follow-up is indicated.

Atrial Myxoma

It is the most frequent benign tumor diagnosed by imaging studies [1,3,4,9]. It is more frequent in women and in adults between 40 and 70 years of age [1,4,9]. It originates from the endocardium of the interatrial septum at the level of the fossa ovalis with endoluminal growth toward the left atrium lumen in 90% of cases and in 10% in the right atrium one [1,3,7]. Most are solitary masses, with cases of multiplicity associated with syndromes such as Carney syndrome and non-syndromic familial myxomas [3,7,9].

They are usually mobile and may prolapse into the ventricle during diastole, producing valvular stenosis. Nonetheless, most are asymptomatic and are detected incidentally. The classic clinical triad of systemic embolisms, fever, and weight loss has been described [4,7].

**Figure 13:**
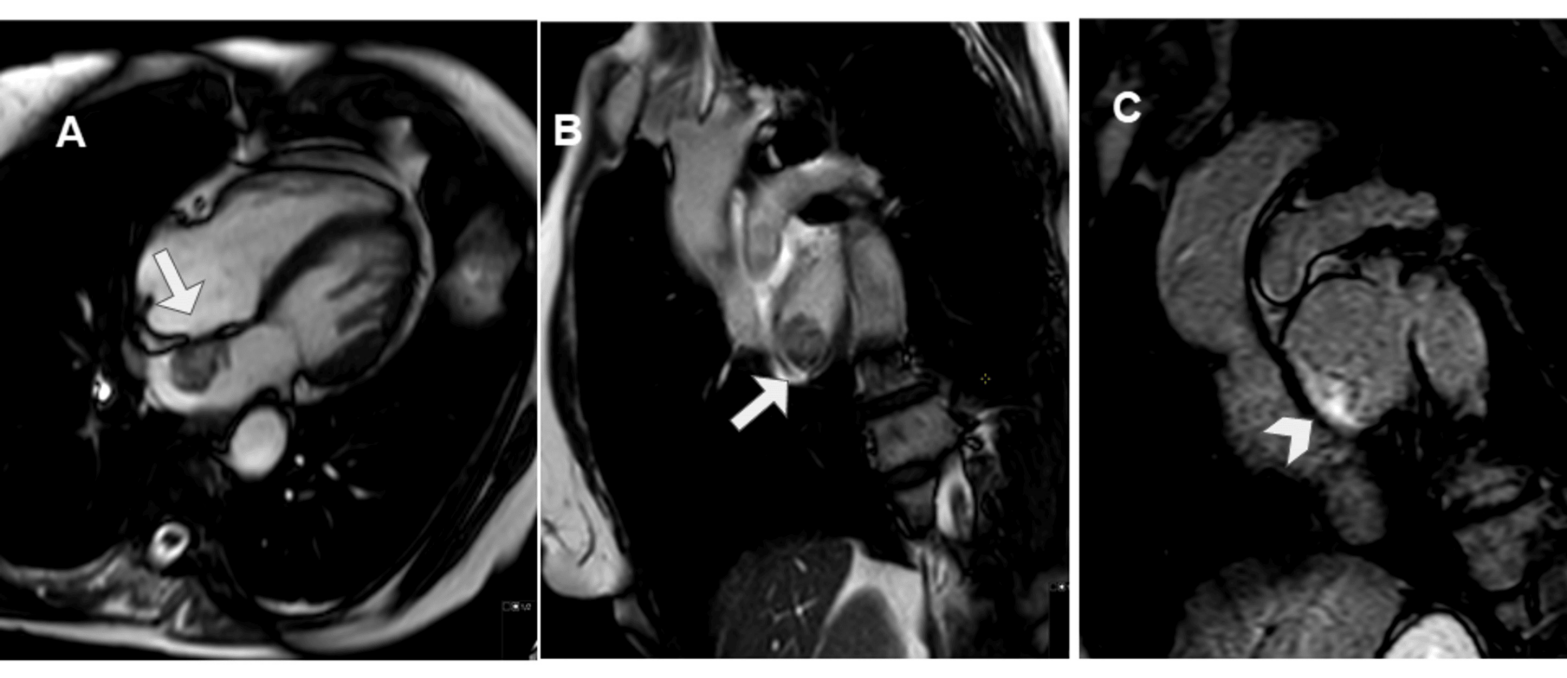
CMR imaging. This shows a hypointense mass in the left atrium in the steady-state free precession sequence (arrow in A and B). On LGE, it shows intense late contrast uptake (arrowhead in C). CMR: cardiac magnetic resonance; LGE: late gadolinium enhancement.

They are usually diagnosed by Echo. This shows a mass of variable size (2-11 cm) with heterogeneous echogenicity, which is mobile and prolapses into the left ventricle. CT without contrast shows a hypodense mass with respect to the myocardium and calcifications, punctate or coarse in 14% of cases [1,7]. On CECT, they show an heterogeneous uptake of contrast. On CMR, they are isointense or hypointense with respect to the myocardium on T1-wTSE and hyperintense on T2-wTSE sequences and hypointense on SSFP cine sequences. They may show foci of signal drop secondary to calcifications or hemosiderin deposits. On gadolinium sequences, they do not show first-pass uptake on perfusion, although they do show heterogeneous late contrast enhancement due to areas of hemorrhage, calcifications, necrosis, or cystic degeneration (Figure 13) [1,7].

The existence of mural tumor thrombi has also been described. On PET-CT, they show very variable tracer uptake, from absent to high uptake [1]. Their main differential diagnosis is with thrombi, due to their frequency and location in the left atrium, which do not show contrast enhancement unlike myxomas [7,9]. Treatment is always surgical to avoid complications, with a good prognosis and a low recurrence rate except in syndromic cases. After treatment, annual echocardiographic follow-up is performed for five years [1,3,7].

Cardiac Lipoma

It is the third most frequent primary cardiac tumor [4,7]. They are small masses that are sessile (epicardial location) or pedunculated (subendocardial) [7], generally originating from the interatrial septum in the right atrium (FIGURE 8) or in the left ventricle [9]. They can cause dyspnea due to outflow tract obstruction or arrhythmias, in which case treatment is surgical. They show imaging characteristics identical to adipose tissue on all imaging modalities: hypodense on CT and hyperintense on T1-wTSE and T2-wTSE sequences, suppressing on fat-saturation sequences, and they usually do not enhance on contrast sequences or enhancement is early or minimal [1,7,9]. If a lesion with fatty content enhances and/or has an infiltrative appearance, the first diagnostic possibility should be liposarcoma [9].

Cardiac Rhabdomyoma

It is the most frequent primary cardiac tumor in children, and most cases are diagnosed in the prenatal or neonatal period [1, 3,9]. Most of them are multiple (60%), both sporadic cases and those associated with tuberous sclerosis. Morphologically, they are intramyocardial ventricular masses that may bulge into the ventricular lumen in a variable degree [1,4].

Most of them are asymptomatic, although some can cause outflow tract obstruction or arrhythmias. They have a good prognosis, since most of them regress spontaneously [1,3,4]. If they persist, treatment is surgical or therapy with mTOR pathway inhibitors (sirolimus, everolimus) [1].

UCG shows intramyocardial hyperechogenic masses, whereas on CMR they are usually masses with signals similar to the myocardium on almost all sequences or slightly hyperintense on T2-wTSE sequences. They appear as masses without uptake contrast on perfusion sequences relative to the myocardium and do not show enhancement on LGE [1,3,9].

Cardiac Fibroma

It is the second most frequent tumor in children after rhabdomyoma [1,3,4,9]. They can be sporadic or associated with Gorlin or Gardner syndromes [1,3,7]. There are asymptomatic cases or may cause arrhythmias and sudden death due to atrioventricular block [1,7].

On UCG, they are intramyocardial masses between 3 and 8 cm in size, akinetic, and located mainly in the free wall of the left ventricle, interventricular septum, and free wall of the right ventricle, in that order [1,4,7,9]. On CT they may have calcifications in up to 50% of cases, although it is more frequent to see them in older adults [3,4,7]. CMR shows well-defined lesions, with a rim of displaced normal myocardium, hypointense on SSFP cine sequences, hypointense on T1-wTSE sequences, and hypointense or isointense on T2-wTSE sequences. On gadolinium sequences, they do not show perfusion uptake but do show late enhancement: homogeneous or peripheral due to perilesional fibrous tissue [1,3,4,7]. On PET-CT they usually have moderate FDG uptake. Treatment is surgical due to the high risk of cardiac arrhythmias, with an excellent long-term outcome even in cases of incomplete resection [6].

Hemangiomas

They account for 5-10% of primary cardiac neoplasms and are more frequent in young people (30-40 years old) and slightly more frequent in women [3,4,9]. They mainly affect the ventricles although they can be located in any cardiac chamber and any myocardial layer [7,9]. They are tumors between 2 and 3.5 cm, sessile or polypoid; isointense or hypointense on T1-wTSE sequences and markedly hyperintense on SSFP cine and T2-wTSE sequences relative to the myocardium. With gadolinium, they show intense uptake on perfusion and intense and heterogeneous uptake on LGE due to the presence of phleboliths [3,7,9]. From an imaging point of view, they can emulate a sarcoma; although in the latter, calcifications are very rare. The great majority are asymptomatic [9]. In symptomatic cases, surgery is the treatment of choice. Presurgical embolization is usually necessary due to the high risk of bleeding [2].

Cardiac Paragangliomas

**Figure 14:**
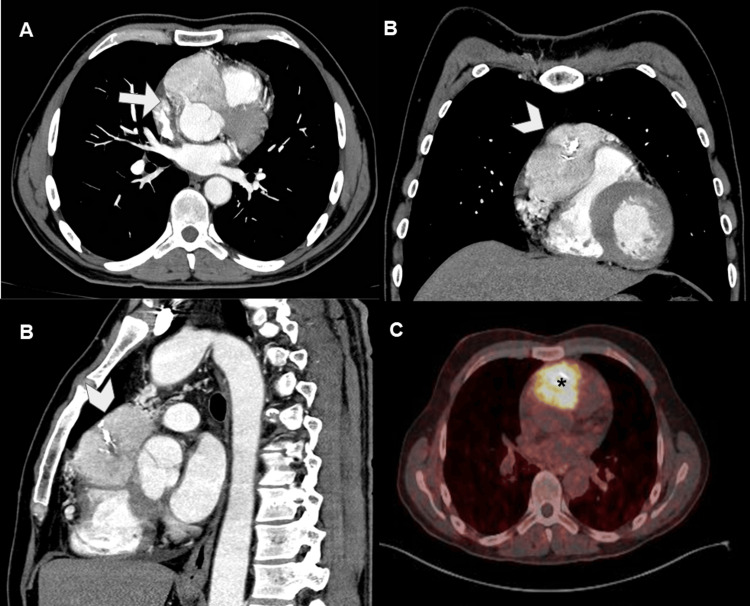
Cardiac paraganglioma in the right atrioventricular sulcus. CECT on axial, coronal, and sagittal reconstructions (A-C) and PET-CT with somatostatin analogue (C). The images show a hyperenhancing mass in the right atrioventricular sulcus and occupying the anterior mediastinum. In the axial view, the mass surrounds the origin of the right coronary artery (arrow in A). Coronal and sagittal views show calcifications within the tumor (arrowhead in B and C). PET-CT shows uptake of the radiotracer by the tumor, demonstrating its neuroendocrine origin (asterisk in D). CECT: contrast-enhanced computed tomography; PET-CT: positron emission tomography-computed tomography.

It is a very rare tumor: 1.5-1.6 per 10,000 people [1,9]. There are sporadic and familial cases (10-30%). They are tumors derived from chromaffin cells of the mediastinum. They present as well-defined multilobulated masses of 3-8 cm affecting the left atrium, ascending aorta, interatrial septum, or atrioventricular groove (Figure 14) [1,7].

Most are secretory and therefore symptomatic with hypertensive crises with very variable periodicity (daily to months). CMR shows a mass that is hypointense on T1-wTSE (except for areas of hemorrhage, which appear hyperintense) and hyperintense on T2-wTSE. They are vascular masses, with vessels usually arising from the right coronary artery. They show intense enhancement on both perfusion and LGE sequences, usually heterogeneous due to hemorrhage and necrosis areas, sometimes giving a stellate appearance [1,7,9]. Nuclear medicine studies with somatostatin analogs are useful for detecting small lesions, multiplicity or distant metastases. They are usually benign but some of them can metastasize. Their treatment is surgical, being complex due to their high degree of vascularization, close relationship with cardiac structures, and risk of hypertensive crises during the surgery. The latter is the reason why biopsy is not recommended [7].

Malignant tumors

They are mainly located in the right chambers and are characterized by being large heterogeneous masses, greater than 5 cm, with a broad implantation base, infiltrative appearance, multichamber involvement, and frequent association with pericardial and/or pleural effusion [1,2,6-9]. These morphological characteristics can be appreciated in imaging studies, especially in CMR, which stands as the most accurate technique for their diagnosis since it can identify areas of necrosis, hemorrhage, and cystic degeneration responsible for their characteristic heterogeneity. In addition, it is characteristic that they show a high degree of contrast enhancement both on perfusion and in LGE sequences [2,7-9]. Some studies even show that the absence of uptake in both contrast sequences virtually excludes the possibility of malignancy [2]. They also show high FDG uptake on PET-CT (at least 3.8 SUV).

Histologically, they can be divided into sarcomas, lymphomas, and mesotheliomas [9]. Among cardiac sarcomas, the most frequent type is angiosarcoma. Less frequent ones include undifferentiated sarcomas, leiomyosarcomas, rhabdomyosarcomas, fibrosarcomas, liposarcomas, and extraskeletal osteosarcomas [9].

Benign tumors, on the contrary, are smaller in size, with a narrow base implantation and therefore a high degree of mobility, more frequently located in left chambers, and they respect adjacent structures. They are not usually associated with pleural nor pericardial effusion. Although some benign masses show contrast enhancement (atrial myxoma or hemangiomas), many do not (lipomas) or it is very slight and late (fibroelastomas, fibromas) [1,2,7,8]. Benign tumors do not show uptake in PET-CT studies except in rare cases (brown fat present in lipomatous hypertrophy of the interatrial septum) (Table 3).

**Table 3:** Comparison of benign and malignant primary cardiac tumors. UCG: echocardiography; CMR: cardiac magnetic resonance.

Characteristic	Benign	Malignant
Frequency	75%	25%
Main representatives	Myxoma (most frequent), lipoma, fibroelastoma	Sarcomas (mainly angiosarcoma), lymphoma
Typical clinical presentation	Asymptomatic or symptoms from valvular obstruction, emboli, arrhythmias	Aggressive symptoms: dyspnea, chest pain, arrhythmias, superior vena cava syndrome, poor general condition
Usual location	Left atrium (myxoma)	Right atrium (angiosarcoma)
Imaging diagnosis	UCG (initially), CMR for characterization	UCG (detection), CT/PET-CT (extension), CMR (tumor characterization and relationship with structures)
Evolution	Generally favorable after complete resection	Rapid growth and infiltration; frequent metastases
Management	Curative surgery (complete resection)	Surgery if possible (rarely complete), chemotherapy and radiotherapy
Prognosis	Excellent after resection	Very poor (average survival <12 months)

Cardiac Angiosarcoma

They are the most frequent primary malignant cardiac tumors (although rhabdomyosarcoma is the most frequent primary malignant tumor in children), accounting for one third of them with an incidence of 0.0001% [1,3-7]. It is more frequent in middle-aged men, with a peak incidence at 40 years [1,3,5,7,9]. They are mainly located in the free wall of the right atrium, characteristically sparing the interatrial septum [3,4,6,7,9]. They are generally of primary etiology, as secondary ones are rarer (after radiotherapy, Li-Fraumeni syndrome or trisomies 17/18).

Morphologically, they present two variants: an infiltrative variant with a strong tendency for pericardial involvement and pericardial effusion of heterogeneous content (hemorrhagic or with tumoral implants), and a mass-type variant that protrudes into the atrial lumen with extensive areas of necrosis (as in our case) and even the possibility of opening into the atrial lumen resulting in an atrial pseudoaneurysm [3,9]. In both forms, infiltration of adjacent structures (tricuspid valve, right ventricle, or venous structures) is frequent.

They are generally symptomatic due to their size and aggressiveness, presenting with heart failure, dyspnea, cough (especially if pulmonary metastases are present), and, in advanced cases, cardiac tamponade and superior vena cava syndrome [1,3,6,7,9]. They have a poor prognosis due to the difficulty of complete resection, frequent presence of metastases at diagnosis (in 50-89%), and radioresistance [3-7,9].

Their definitive diagnosis is made by biopsy, although imaging studies, especially CMR, have shown high correlation in differentiating benign from malignant neoplasms and can even suggest the diagnosis of sarcomatous lesions. UCG shows them as an infiltrative mass of the left atrium with very heterogeneous echogenicity occupying most of the lumen. In CMR, their appearance depends on their morphological pattern: On one hand the infiltrative variant diffusely affects the left atrium and is associated with heterogeneous signal pericardial effusion (on T1-wTSE sequences, hyperintense areas corresponding to hemorrhage and intermediate signal areas corresponding to tumor implants). On the other hand, the mass-type variant shows a heterogeneous signal on T1-wTSE sequences with hypointense areas corresponding to necrosis, intermediate signals corresponding to tumor, and hyperintense areas corresponding to hemorrhage [1,3,7,9]. On T2-wTSE, the signal is heterogeneous although predominantly hyperintense. After gadolinium administration, the mass-type variant shows extensive areas of no enhancement corresponding to necrosis and nodular areas with enhancement of viable tumor [1,7], as in our case. Meanwhile, in the infiltrative form, enhancement is heterogeneous and may present a sunray pattern due to the presence of linear enhancement of vascular structures crossing the pericardium [3,4,7,9]. CT shows a very heterogeneous mass consistent with the previously described tissue changes of the tumor and does not present calcifications [3,4]. PET-CT shows high FDG uptake and is useful for staging. The most affected organs by metastases, in order of frequency, are lung, liver, mediastinal lymph nodes, bone, adrenals, and spleen [3].

The differential diagnosis includes vascularized cardiac lesions such as cardiac hemangiomas (in which phleboliths are frequent) and other types of cardiac sarcomas, although these are much rarer and affect the left chambers mostly [1].

Macroscopically, these tumors present a firm, heterogeneous consistency with reddish-violet areas corresponding to highly vascularized regions. They exhibit poorly defined margins with an infiltrative appearance into adjacent structures. Extensive areas of hemorrhage and necrosis are common, as depicted in our case, and allowed distinguishing it from other primary cardiac tumors, such as myxomas, which are typically more circumscribed and gelatinous, or rhabdomyosarcomas, which are generally less hemorrhagic [10].

Histologically, the predominant pattern consists of spindle cells (the pattern of our case), although epithelioid or mixed forms may also be observed. A hallmark feature is the presence of anastomosing vascular channels formed by endothelial cells showing marked nuclear atypia, frequent mitoses, and poorly differentiated solid areas. Irregular vascular spaces filled with blood and lined by neoplastic cells are also characteristic [10].

Immunohistochemically, the endothelial origin is confirmed by positivity for CD34 and CD31 [7,10]. ERG is the most sensitive and specific marker, with diffuse positivity in nearly all cases, allowing for a definitive diagnosis. Additional findings include positivity for FLI-1 and a Ki-67 proliferation index ranging from 10-30%, with values above 10% indicating poor prognosis [7,10]. In contrast, these tumors are negative for p53, CD68, SMA, HMB-45, CK, MelanA, and LCA [7]. Overexpression of MDM2 in intimal sarcoma and C-MYC amplification in certain angiosarcomas, particularly secondary forms, are useful features for differential diagnosis [10].

Treatment is complex and multidisciplinary, as there is no standard therapy, mainly due to their low tumor incidence and the limited effectiveness of current treatments [5,6], with an overall survival of 11-14 months in localized tumors and six months in advanced cases [3,5-7]. In local stages, treatment is based on radical surgery with complete tumor resection, possibly including adjuvant radiotherapy. Neoadjuvant chemotherapy is also possible to increase resectability and survival [6,7].

Other Sarcomas

Undifferentiated pleomorphic sarcoma represents the second most frequent cardiac sarcoma, although much rarer than angiosarcoma [1,9]. It is more frequent in adults aged 40-50 years and typically occurs in the left atrium. It is a large infiltrative mass in the posterior wall of the left atrium with frequent involvement of the pulmonary veins [3,4]. On CMR, it shows an isointense signal compared to myocardium in T1-w and T2-wTSE sequences and heterogeneous contrast uptake both in perfusion and LGE. The differential diagnosis is made with angiosarcoma when located in the left atrium and atrial myxoma [1].

Leiomyosarcoma: Polylobulated masses with broad implantation base and infiltrative appearance in the left atrium, with possible associated calcifications. They are multiple in 30% of cases [1,9]. On CMR, they are hypointense in T1-wTSE and hyperintense in T2-wTSE sequences, and after gadolinium administration, they show heterogeneous enhancement [1,9].

Other sarcomatous histological types derived from the cardiac stroma, even less frequent than those previously mentioned, include liposarcoma, rhabdomyosarcoma, and synovial sarcoma, except for rhabdomyosarcoma, which is the most frequent malignant neoplasm in children [9]. These masses do not show preference for any specific cardiac chamber and share general common characteristics with other sarcomas [3,7]. Their prognosis is poor, with survival less than one year. The main causes of death are heart failure, cardiac tamponade, or arrhythmias [7].

Cardiac Mesotheliomas

They represent half of the primary pericardial tumors and are not associated with asbestos exposure as pleural mesotheliomas. On CMR, they show marked thickening of both pericardial layers with isointense signal in T1-wTSE sequences, hyperintense in T2-wTSE, and show intense contrast enhancement after gadolinium administration. In addition, they do not usually invade the cardiac wall [9].

Primary Cardiac Lymphoma

Primary cardiac lymphoma represents only 1% of all primary cardiac tumors, with secondary cardiac lymphomatous involvement being much more frequent [1,4,7,9]. It is more frequent in men aged 60-70 years and in immunosuppressed individuals [3]. These are aggressive non-Hodgkin type lymphomas [1]. Its most frequent origin is the atrioventricular groove with secondary involvement of the right atrium and ventricle in 92% of cases [3,7,9]. Pericardial involvement is frequent, sometimes being its only form of presentation [9].

In imaging, it can present three morphologic patterns: infiltrative mass, intracavitary mass, and multiple nodules. The mass-type form bulges into the right atrial and ventricular lumen, with valvular involvement being rare [3,7]. The infiltrative form surrounds the coronary arteries and the cardiac vascular pedicle (aorta and pulmonary arteries). In all three patterns, CMR shows homogeneous tumor tissue (necrosis and bleeding are rare) and isointense or hypointense signal in T1-w and T2-wTSE sequences, with heterogeneous enhancement after gadolinium administration (in both perfusion and LGE) [3,7]. PET-CT is useful for diagnosis and follow-up, revealing marked FDG uptake with an SUV greater than 16 [1]. Treatment consists of chemotherapy with anthracyclines and Rituximab. Prognosis is poor with a mean overall survival of 63 months at five years [3].

Cardiac Metastases

They are the most frequent tumors, 20 to 40 times more frequent than primary tumors [4,9]. The possible routes of dissemination are direct extension, hematogenous, lymphatic, and venous. They can have an epicardial, intramyocardial, or intracavitary location [1,7,9]. Among them, the most frequent are lymphatic spread and direct extension from a thoracic neoplasm, usually from a pulmonary tumour. The most frequent primary tumors correspond to lung, breast, melanoma, lymphoma, and leukemia [1,9]. Melanoma is notable for its tendency to metastasize to myocardium.

In imaging, pericardial metastases manifest as pericardial effusion, diffuse thickening of the serosa, implants, or both. Myocardial metastases generally affect the lateral wall of the left ventricle and interventricular septum. Endocavitary metastases usually appear in the context of venous dissemination in the form of tumor thrombus, generally from pulmonary neoplasm with cardiac involvement by direct extension [9]. On CMR, metastasis shows isointense signal in T1-wTSE sequences, slightly hyperintense in T2-wTSE, and heterogeneous contrast enhancement. On T1-wTSE sequence, melanoma or hemorrhagic metastases show hyperintense signal. Tumor thrombus show contrast uptake unlike non-tumor thrombus which do not [7,9].

## Conclusions

Primary cardiac tumors are very rare entities, with a prevalence of approximately 0.001-0.03% in autopsy series. Of these, about 75% correspond to benign tumors and 25% to malignant tumors.

Among benign tumors, atrial myxoma is the most frequent in clinical practice, mainly located in the left atrium. Clinically, these tumors may remain asymptomatic or manifest with obstructive symptoms (valvular obstruction, heart failure), systemic emboli, or arrhythmias. Diagnosis is initially established by UCG, while CMR allows morphological and tissue characterization of the mass, as well as its relationship with adjacent structures. The treatment of choice is complete resection, with an excellent prognosis in most cases.

In contrast, malignant cardiac tumors are extremely rare, with sarcomas being the most representative, particularly angiosarcoma, which is most frequently located in the right atrium. These tumors are characterized by aggressiveness and tendency to infiltrate structures and early distant dissemination. Clinical presentation is usually evident with dyspnea, chest pain, arrhythmias, superior vena cava syndrome, and systemic symptoms. Diagnosis requires a multimodal approach: UCG is the initial study for detection, while CT and PET-CT allow assessment of extracardiac extension. CMR is the most accurate study to differentiate malignant masses from thrombi or pseudotumors. Standard treatment includes surgical resection associated with chemotherapy, although in most cases complete resection is not possible. Prognosis is unfavorable, with mean survival less than 12 months.

The presented case, an 83-year-old woman with a right atrial angiosarcoma, illustrates the aggressive nature of these neoplasms and the importance of clinical suspicion, the use of advanced imaging techniques for diagnosis, as well as the therapeutic limitations and poor prognosis of malignant cardiac tumors.
